# Supplementary material for: Association of a healthy beverage score with total mortality in the adult population of Spain: A nationwide cohort study
Source: PLoS Med. 2024 Jan 23;21(1):e1004337. doi: 10.1371/journal.pmed.1004337 (PMC10805278; doi:10.1371/journal.pmed.1004337)
Supplement: S1 STROBE Checklist — (DOCX) [file pmed.1004337.s001.docx]

**S1 STROBE Checklist.**

|  |  | | | **Recommendation** |  |  |
| --- | --- | --- | --- | --- | --- | --- |
| **Title and abstract** | | | | | |  |
|  | 1 | | | (*a*) Indicate the study’s design with a commonly used term in the title or the abstract | Title; Abstract [Methods and Findings, paragraph 1] |  |
|  |  |  |  | (*b*) Provide in the abstract an informative and balanced summary of what was done and what was found | Abstract [Methods and Findings, paragraphs 1-2] |  |
| **Introduction** | | | | |  |  |
| Background/  rationale | 2 | | | Explain the scientific background and rationale for the investigation being reported | Abstract [Background, paragraph 1]; Author summary [Why Was This Study Done?, bullet points 1-4]; Introduction [paragraphs 1-3] |  |
| Objectives | 3 | | | State specific objectives, including any pre-specified hypotheses | Abstract [Background, paragraph 1]; Introduction [paragraph 4] |  |
| **Methods** | | | | |  |  |
| Study design | 4 | | | Present key elements of study design early in the paper | Abstract [Methods and findings, paragraph 1]; Methods [Study design and participants, paragraphs 1-5] |  |
| Setting | 5 | | | Describe the setting, locations, and relevant dates, including periods of recruitment, exposure, follow-up, and data collection | Methods [Study design and participants, paragraph 1; Study variables, paragraph 1; Mortality assessment, paragraph 1] |  |
| Participants | 6 | | | (*a*) *Cohort study*—Give the eligibility criteria, and the sources and methods of selection of participants. Describe methods of follow-up  *Case-control study*—Give the eligibility criteria, and the sources and methods of case ascertainment and control selection. Give the rationale for the choice of cases and controls  *Cross-sectional study*—Give the eligibility criteria, and the sources and methods of selection of participants | Methods [Study design and participants, paragraph 1-2; Mortality assessment, paragraph 1] |  |
|  |  |  |  | (*b*)*Cohort study*—For matched studies, give matching criteria and number of exposed and unexposed  *Case-control study*—For matched studies, give matching criteria and the number of controls per case | Not applicable. |  |
| Variables | 7 | | | Clearly define all outcomes, exposures, predictors, potential confounders, and effect modifiers. Give diagnostic criteria, if applicable | Methods [Study variables: Dietary history, paragraph 1]; Methods [Study variables: Healthy Beverage Score, paragraph 1]; Methods [Study variables: Mortality assessment, paragraph 1]; Methods [Study variables: Confounders, paragraph 1]; Methods [Statistical methods, paragraphs 1-2] |  |
| Data sources/ measurement | 8* | | | For each variable of interest, give sources of data and details of methods of assessment (measurement). Describe comparability of assessment methods if there is more than one group | The variable of interest in this study was total mortality. All the sources and details of the data are provided in the manuscript [See Methods section] |  |
| Bias | 9 | | | Describe any efforts to address potential sources of bias | Selection bias was assessed in the sampling methodology, in addition to imputation for missing data. In addition, classification bias was assessed using the validity of the beverage measurement tool (HD-ENRICA). Methods [Study variables: Dietary history, paragraph 1]; Methods [Study variables: Healthy Beverage Score, paragraph 1] |  |
| Study size | 10 | | | Explain how the study size was arrived at | This is a multipurpose cohort. The sample size was nationally representative for Spain. Methods [Study variables: Dietary history, paragraph 1]. The sample size of the ENRICA study allows to estimate the prevalence of a risk factor with a 95% confidence interval of ±1% assuming a risk factor prevalence of 50% and a sampling design effect of 1.3. |  |
| Quantitative variables | 11 | | | Explain how quantitative variables were handled in the analyses. If applicable, describe which groupings were chosen and why | Quantitative variables were not categorized. |  |
| Statistical methods | 12 | | | (*a*) Describe all statistical methods, including those used to control for confounding | Methods [ Study variables: Confounders, paragraph 1]; Methods [Statistical analysis: paragraphs 1-3] |  |
|  |  |  |  | (*b*) Describe any methods used to examine subgroups and interactions | Methods [Statistical analysis: paragraph 2] |  |
|  |  |  |  | (*c*) Explain how missing data were addressed | Methods [Study variables: Confounders, paragraph 2] |  |
|  |  |  |  | (*d*) *Cohort study*—If applicable, explain how loss to follow-up was addressed  *Case-control study*—If applicable, explain how matching of cases and controls was addressed  *Cross-sectional study*—If applicable, describe analytical methods taking account of sampling strategy | In this study, there was no loss to follow-up due to linkage with the National Death Index. Methods [Mortality assessment, paragraph 1] |  |
|  |  |  |  | (*e*) Describe any sensitivity analyses | Sensitivity analyses were performed excluding deaths in the first 3 years of follow-up and individual items of the HBS were assessed with adjustment for the remaining items that are part of the score: Methods [Statistical analysis, paragraph 2] |  |
| **Results** | | | | |  |  |
| Participants | | 13* | (a) Report numbers of individuals at each stage of study—eg numbers potentially eligible, examined for eligibility, confirmed eligible, included in the study, completing follow-up, and analysed | | Methods [Study design and participants: paragraph 2]; Results [paragraph 1] |  |
|  |  |  | (b) Give reasons for non-participation at each stage | | Methods [Study design and participants, paragraph 1] |  |
|  |  |  | (c) Consider use of a flow diagram | | Please, see S1 Fig. |  |
| Descriptive data | | 14* | (a) Give characteristics of study participants (eg demographic, clinical, social) and information on exposures and potential confounders | | Results [paragraph 1-2] |  |
|  |  |  | (b) Indicate number of participants with missing data for each variable of interest | | Methods [Study variables: Confounders, paragraph 2] |  |
|  |  |  | (c) *Cohort study*—Summarise follow-up time (eg, average and total amount) | | Follow-up time (years) was summarised as mean, SD and range. Results [paragraph 3]. |  |
| Outcome data | | 15* | *Cohort study*—Report numbers of outcome events or summary measures over time | | Results on all-cause mortality were reported. Results [paragraph 3] |  |
|  |  |  | *Case-control study—*Report numbers in each exposure category, or summary measures of exposure | | Not applicable |  |
|  |  |  | *Cross-sectional study—*Report numbers of outcome events or summary measures | | Not applicable |  |
| Main results | | 16 | (*a*) Give unadjusted estimates and, if applicable, confounder-adjusted estimates and their precision (eg, 95% confidence interval). Make clear which confounders were adjusted for and why they were included | | Methods [Statistical analysis, paragraph 2]; Results [Paragraph 3]. S3 Table; S1 Fig; S2 Fig. |  |
|  |  |  | (*b*) Report category boundaries when continuous variables were categorized | | Methods [Statistical analysis, paragraph 2]; Results [Table 2, Table 5] |  |
|  |  |  | (*c*) If relevant, consider translating estimates of relative risk into absolute risk for a meaningful time period | | Abstract [Methods and Findings, paragraph 2]; Results [paragraph 3] |  |
| Other analyses | | 17 | Report other analyses done—eg analyses of subgroups and interactions, and sensitivity analyses | | Adjusted and unadjusted results from analyses of interactions and sensitivity analysis [Results paragraph 3] were included (also see Results [Table 2, Table 5, S3 Table, Figure 2, S1 Fig, S2 Fig] |  |
| **Discussion** | | | | |  | |
| Key results | | 18 | Summarise key results with reference to study objectives | | Discussion [paragraph 1] |  |
| Limitations | | 19 | Discuss limitations of the study, taking into account sources of potential bias or imprecision. Discuss both direction and magnitude of any potential bias | | Discussion [paragraph 10] |  |
| Interpretation | | 20 | Give a cautious overall interpretation of results considering objectives, limitations, multiplicity of analyses, results from similar studies, and other relevant evidence | | Discussion [paragraph 9] |  |
| Generalisability | | 21 | Discuss the generalisability (external validity) of the study results | | Conclusion [paragraph 8] |  |
| **Other information** | | | | |  |  |
| Funding | | 22 | Give the source of funding and the role of the funders for the present study and, if applicable, for the original study on which the present article is based | | Submission form |  |
