# Supplementary material for: Association of a healthy beverage score with total mortality in the adult population of Spain: A nationwide cohort study
Source: PLoS Med. 2024 Jan 23;21(1):e1004337. doi: 10.1371/journal.pmed.1004337 (PMC10805278; doi:10.1371/journal.pmed.1004337)
Supplement: S1 Text — (DOCX) [file pmed.1004337.s002.docx]

**S1 Text. Prespecified analysis plan and modifications**

**Study objective**

To assess the association between a Healthy Beverage Score (HBS) and total mortality in the ENRICA cohort, a representative sample from the adult Spanish population.

**Participants**

Data were obtained from the Study on Nutrition and Cardiovascular Risk in Spain (ENRICA) cohort. In brief, 13,105 individuals aged 18 years and older were recruited during 2008 to 2010. A stratified cluster sampling was performed to guarantee the representativeness of the sample. Three sequential stages were followed for data collection. First, sociodemographic, lifestyle characteristics, and morbidity information was obtained through a telephone interview. Second, blood and urine samples were collected in a first home visit. Third, a physical examination and a face-to-face dietary history (DH-ENRICA) were completed in a second home visit. The rate response was 51 %.

Participants without dietary information and with implausible values for total energy intake (<800 kcal/day or >5000 kcal/day in men; <500 kcal/day or >4000 kcal/day in women) will be excluded from the analyses.

**Dietary information and the Healthy Beverage Score (HBS)**

Dietary information was obtained with the DH-ENRICA, a computerized dietary history tool. Certified non-medical interviewers administered the tool, which systematically compiled data on 861 distinct food items, including 82 varieties of beverages. Participants were required to report all consumables, both food and beverages, ingested at least biweekly over the preceding year, inclusive of both weekdays and weekends. To measure portion sizes in grams per day, we used a wide range of tools, including 127 digitized images, household measurements, and standard proportions derived from typical Spanish recipes. We estimated beverage consumption using 14 digitized images and 23 household measurements.

The dietary data were used to calculate a 7-item HBS based on a previous one described by Hu et al [1]. A modification was made to the classification of the alcohol consumption item by considering no or moderate alcohol consumption as healthy.

**Assessment of mortality**

The study assessed mortality by linking participant data to the Spanish National Death Index. Participants were monitored continuously from 2008-2010 until 31 January 2022. The study ended when participants died or when the follow-up period ended, whichever came first.

**Statistical analyses**

Multiple imputation will be used to handle missing data. The Hazard Ratio and 95% Confidence Interval will be calculated using Cox proportional hazard models. The Schoenfield residuals will be used to check the proportional hazard assumption. Statistical analyses will be conducted using Stata version 17.

**Confounders:** The confounders were selected based on literature and a previous study on the association of a HBS and age-related frailty [2] as follows:

- Age (years, continuous)
- Sex (men, women)
- Educational level (primary or less, secondary, university)
- Smoking (non-smoker, former smoker, current smoker)
- BMI (<25, ≥25 and ≤30, >30 kg/m^2^)
- Time watching TV (hours, continuous)
- Physical activity (METs-h/week, continuous)
- Energy intake (kcal/day, continuous)
- Mediterranean diet without including alcohol (maximum=8)
- Fiber intake (g/d continuous)
- Fruit and vegetable consumption (g/d, continuous)
- Hypertriglyceridemia (yes/no)
- Hypercholesterolemia (yes/no)
- Hypertension (yes/no)
- Number of chronic conditions (0, 1, and ≥2)
- Number of medications (0, 1–3, >3)

Models will be adjusted as follows:

- Model 1 will be adjusted for age and sex.
- Model 2 will be adjusted for variables in model 1 plus educational level, smoking status, body mass index (BMI), physical activity in leisure time, total energy intake, fruits and vegetables consumption, total fiber intake, hypertriglyceridemia, hypercholesterolemia, high blood pressure, number of self-reported chronic conditions and number of medications.
- Model 3 will be adjusted for factors in Model 2 plus adherence to the Mediterranean diet without including alcohol (maximum score = 8), and excluding fruit, vegetable, and fiber consumption.

**Sensitivity analyses**

To assess the potential impact of reverse causality bias, the first 3 years of follow-up will be excluded from the analysis. Also, individual items of the HBS will be assessed according to Model 3.

**Subgroup analyses**

To test for interactions, several analyses will be performed including: age (<60 years; ≥60 years), sex (men; women), BMI (<25, ≥25 and ≤30, >30 kg/m^2^), physical activity (≤ median 61.5 METs-h/wk; > median 61.5 METs-h/wk), and adherence to the Mediterranean diet without alcohol (≤ median 4; > median 4). Multiplicative terms will be included in Model 3.

**Ethics and data protection**

The Clinical Research Ethics Committees of the La Paz University Hospital in Madrid approved the study protocol. All participants provided a written informed consent.

**Modifications to the original plan:**

Based on the reviewers' recommendations, the analysis plan has been revised.

The main changes include using age as the underlying time metric for mortality analyses, providing unadjusted analyses, and conducting an interaction analysis for vegetable consumption and prevalent diseases.

**References**

1. Hu EA, Anderson CAM, Crews DC, Mills KT, He J, Shou H, et al. A Healthy Beverage Score and Risk of Chronic Kidney Disease Progression, Incident Cardiovascular Disease, and All-Cause Mortality in the Chronic Renal Insufficiency Cohort. Curr Dev Nutr. 2020;4(6):nzaa088.

2. Dominguez LJ, Donat-Vargas C, Banegas JR, Barbagallo M, Rodríguez-Artalejo F, Guallar-Castillón P. Adherence to a Healthy Beverage Score Is Associated with Lower Frailty Risk in Older Adults. Nutrients. 2022 Sep 1;14(18):3861.
