# Supplementary material for: Association of a healthy beverage score with total mortality in the adult population of Spain: A nationwide cohort study
Source: PLoS Med. 2024 Jan 23;21(1):e1004337. doi: 10.1371/journal.pmed.1004337 (PMC10805278; doi:10.1371/journal.pmed.1004337)
Supplement: S1 Table — (DOCX) [file pmed.1004337.s003.docx]

**S1 Table. Sex-specific cut-off points for individual items of the Healthy Beverage Score (HBS) in the ENRICA Study (2008-2010) (N=12,161).**

| **Components of the HBS** | | | |  | |  | |  | |
| --- | --- | --- | --- | --- | --- | --- | --- | --- | --- |
|  | | **Minimum score** | |  | |  | | **Maximum score** | |
| **Adequacy** | |  | |  | |  | |  | |
|  | **Low-fat milk (mL/d)** | | |  | |  | |  | |
|  |  | 1 (No consumption) | | 2 (Tertile 1 among consumers) | | 3 (Tertile 2 among consumers) | | 4 (Tertile 3 among consumers) | |
|  |  | 0 | | Male | Female | Male | Female | Male | Female |
|  |  |  |  | > 0 to < 150 | > 0 to < 150 | ≥ 150 to < 228 | ≥ 150 to < 228.6 | ≥ 228 | ≥ 228.6 |
|  | **Coffee and tea (mL/d)** | | |  | |  | |  | |
|  |  | 1 (Quartile 1) | | 2 (Quartile 2) | | 3 (Quartile 3) | | 4 (Quartile 4) | |
|  |  | Male | Female | Male | Female | Male | Female | Male | Female |
|  |  | ≤ 7.3 | ≤ 14.3 | > 7.3 to < 75 | > 14.3 to < 100 | ≥ 75 to < 150 | ≥ 100 to < 187.5 | ≥ 150 | ≥ 187.5 |
| **Moderation** | |  | |  | |  | |  | |
|  | **Whole-fat milk (mL/d)** | | |  | |  | |  | |
|  |  | 1 (Tertile 3 among consumers) | | 2 (Tertile 2 among consumers) | | 3 (Tertile 1 among consumers) | | 4 (No consumption) | |
|  |  | Male | Female | Male | Female | Male | Female | 0 | |
|  |  | > 0 to < 36.9 | > 0 to < 21.4 | ≥ 36.9 to < 150 | ≥ 21.4 to < 100 | ≥ 150 | ≥ 100 |  |  |
|  | **Fruit juice (mL/d)** | | |  | |  | |  | |
|  |  | 1 (Any consumption) | | -- | | -- | | 4 (No consumption) | |
|  |  | Male | Female |  |  |  |  | 0 | |
|  |  | > 0 | > 0 |  | |  | |  |  |
|  | **Artificially sweetened beverages (mL/d)** | | | | |  | |  | |
|  |  | 1 (Any consumption) | | -- | | -- | | 4 (No consumption) | |
|  |  | Male | Female |  |  |  |  | 0 | |
|  |  | > 0 | > 0 |  | |  | |  |  |
|  | **Sugar-sweetened beverages (mL/d)** | | | | |  | |  | |
|  |  | 1 (Tertile 3 among consumers) | | 2 (Tertile 2 among consumers) | | 3 (Tertile 1 among consumers) | | 4 (No consumption) | |
|  |  | Male | Female | Male | Female | Male | Female | 0 | |
|  |  | > 0 to < 42.9 | > 0 to < 28.6 | ≥ 42.9 to < 171.4 | ≥ 28.6 to < 132.9 | ≥ 171.4 | ≥ 132.9 |  |  |
|  | **Alcohol (g/d)** | | |  | |  | |  | |
|  |  | 1 (Heavy drinkers) | | -- | | -- | | 4 (No consumption or moderate drinkers) | |
|  |  | Male | Female |  | |  | | Male | Female |
|  |  | ≥ 40 | ≥ 24 |  | |  | | 0 to < 40 | 0 to < 24 |
