# Supplementary material for: Association of a healthy beverage score with total mortality in the adult population of Spain: A nationwide cohort study
Source: PLoS Med. 2024 Jan 23;21(1):e1004337. doi: 10.1371/journal.pmed.1004337 (PMC10805278; doi:10.1371/journal.pmed.1004337)
Supplement: S2 Table — (DOCX) [file pmed.1004337.s004.docx]

**S2 Table. Mortality risk according to quartiles of the adherence to the Healthy Beverage Score (HBS) in the ENRICA Study from baseline (2008-2010) to January 2022 (N=12,161) excluding the first 3 years of follow-up.**

| **Total mortality** | **Quartile 1**  **HR (95% CI)**  **(Less healthy)** | **Quartile 2**  **HR (95% CI)** | **Quartile 3**  **HR (95% CI)** | **Quartile 4**  **HR (95% CI)**  **(Healthier)** | **p for linear trend**^d^ |
| --- | --- | --- | --- | --- | --- |
| **Deaths, n** | 124/2,796 | 201/2,959 | 203/2,720 | 319/3,566 |  |
| **Person-years** | 26,806 | 28,148 | 24,242 | 32,660 |  |
| **Model 1^a^** | 1 (ref.) | 0.89 [0.69,1.16] | 0.85 [0.65,1.11] | 0.74 [0.59,0.94] | 0.007 |
| **Model 2^b^** | 1 (ref.) | 0.81 [0.62,1.06] | 0.77 [0.59,1.01] | 0.72 [0.56,0.92] | 0.013 |
| **Model 3^c^** | 1 (ref.) | 0.81 [0.62,1.06] | 0.78 [0.60,1.02] | 0.72 [0.56,0.92] | 0.015 |

^a^ Model 1 was an unadjusted model. Age was the underlying time metric.

^b^ Model 2 was adjusted for age (years, continuous), sex (male, female), educational level (primary or less, secondary, university), smoking (non-smoker, former smoker, current smoker), ex-drinker (yes/no), BMI (<25, ≥25 and ≤30, >30 kg/m^2^), time watching TV (hours, continuous), physical activity (METs-hour/week, continuous), energy intake (kcal/day, continuous), fiber intake (g/d continuous), fruit and vegetable consumption (g/d, continuous), hypertriglyceridemia (yes/no), hypercholesterolemia (yes/no), hypertension (yes/no), number of chronic conditions (0, 1, and ≥2), and number of medications (0, 1–3, >3). Age was the underlying time metric.

^c^ Model 3 was adjusted for factors in Model 2 plus adherence to the Mediterranean diet without including alcohol (maximum score=8) and excluding fruit, vegetable, and fiber consumption. Age was the underlying time metric.

^d^ p value for quartile 4 vs quartile 1: Model 1 p=0.014, Model 2 p=0.009; Model 3 p=0.010.

HR, Hazard Ratio; CI, Confidence Interval.
