# Supplementary figures and images for: Association of a healthy beverage score with total mortality in the adult population of Spain: A nationwide cohort study
Source: PLoS Med. 2024 Jan 23;21(1):e1004337. doi: 10.1371/journal.pmed.1004337 (PMC10805278; doi:10.1371/journal.pmed.1004337)

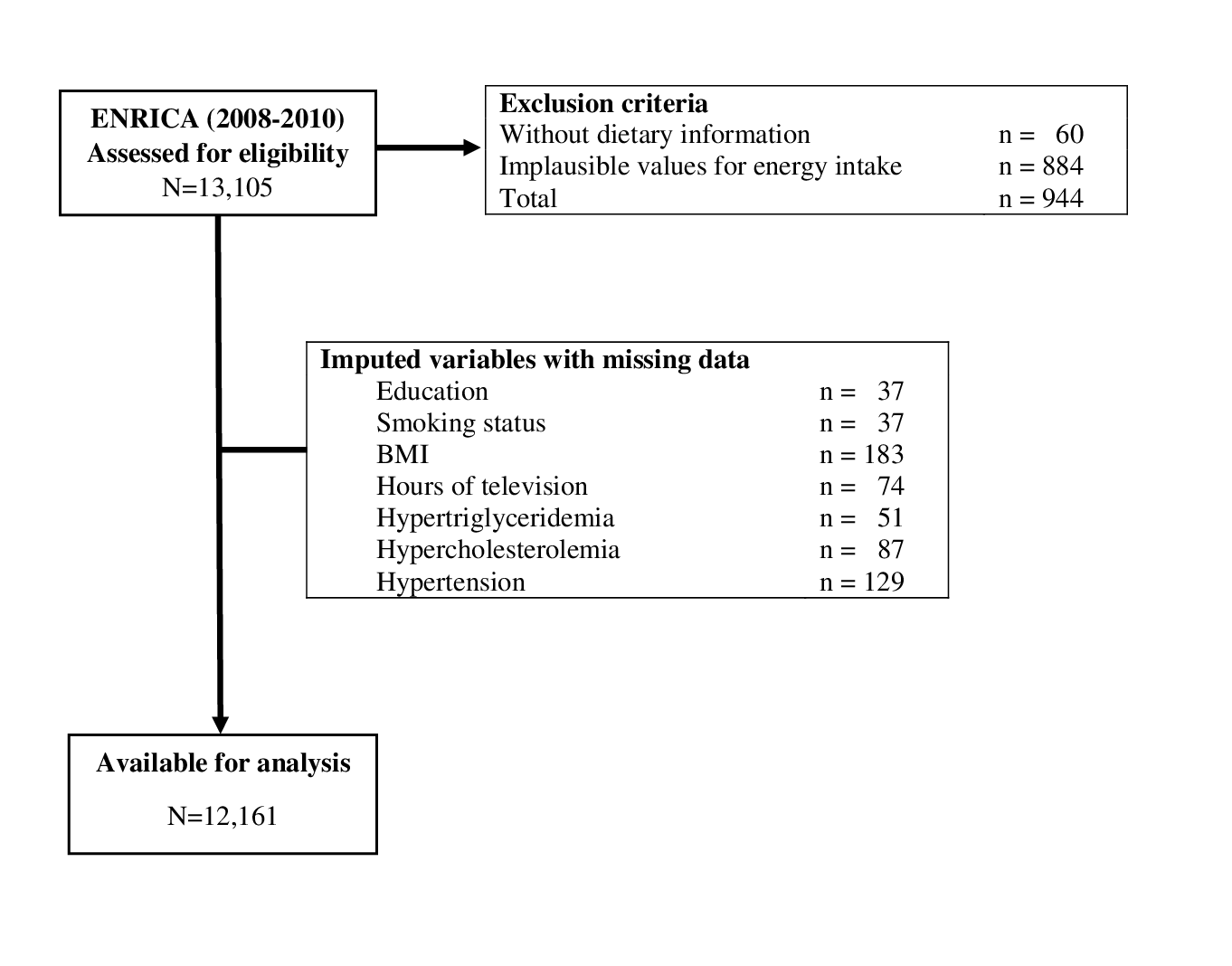

Supplement: S1 Fig — (TIF) [file pmed.1004337.s005.tif]

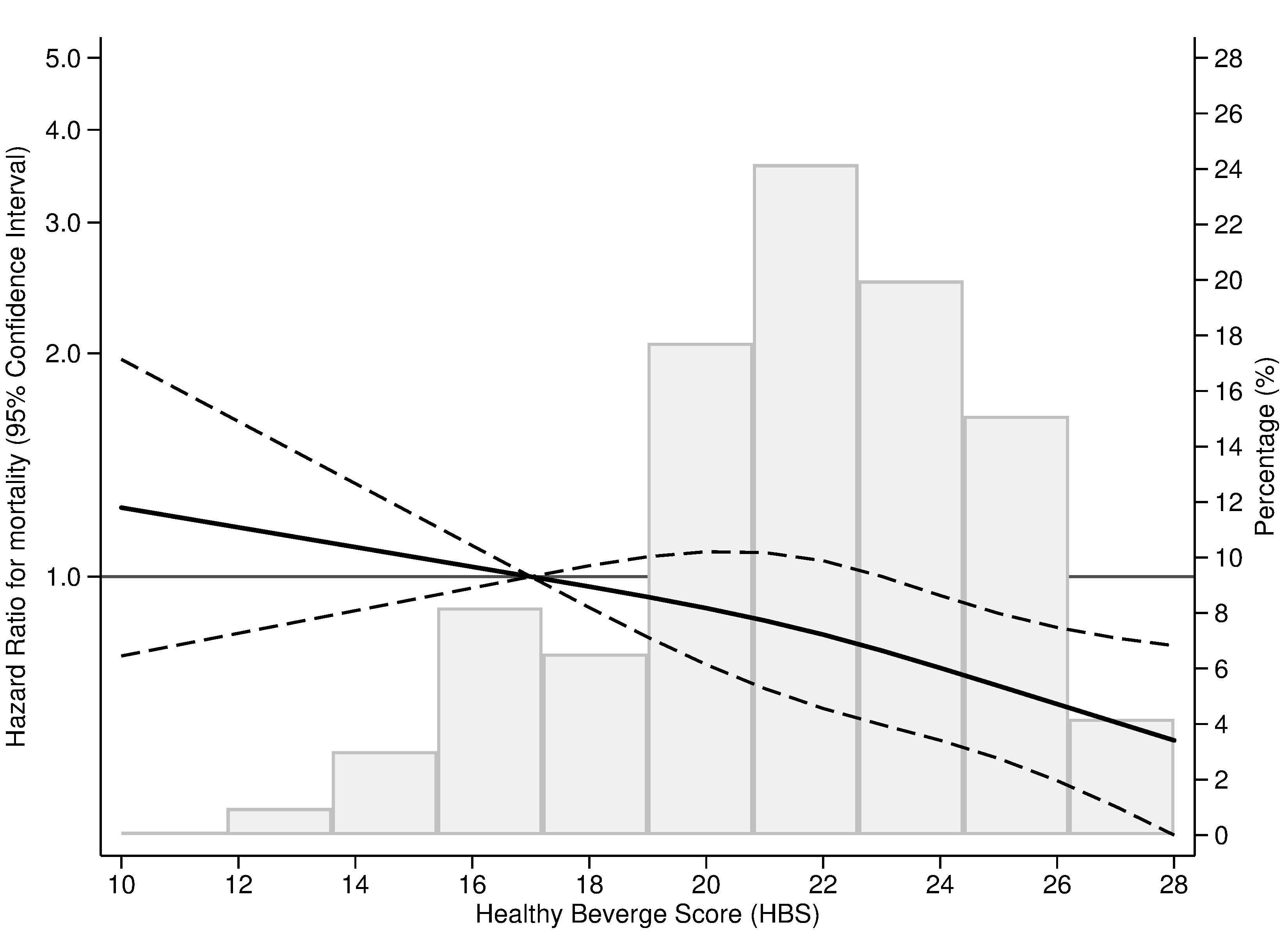

Supplement: S2 Fig — Lines are restricted cubic splines, showing the dose-response association of the Healthy Beverage Score (HBS) with mortality. The solid line represents the hazard ratio (HR), and the dashed lines indicate the lower and upper 95% confidence intervals. The knots were located at the 10th, 50th, and 90th percentiles (corresponding to HBS scores 17, 22 and 25, respectively). p for non-linearity = 0.003. (TIF) [file pmed.1004337.s006.tif]

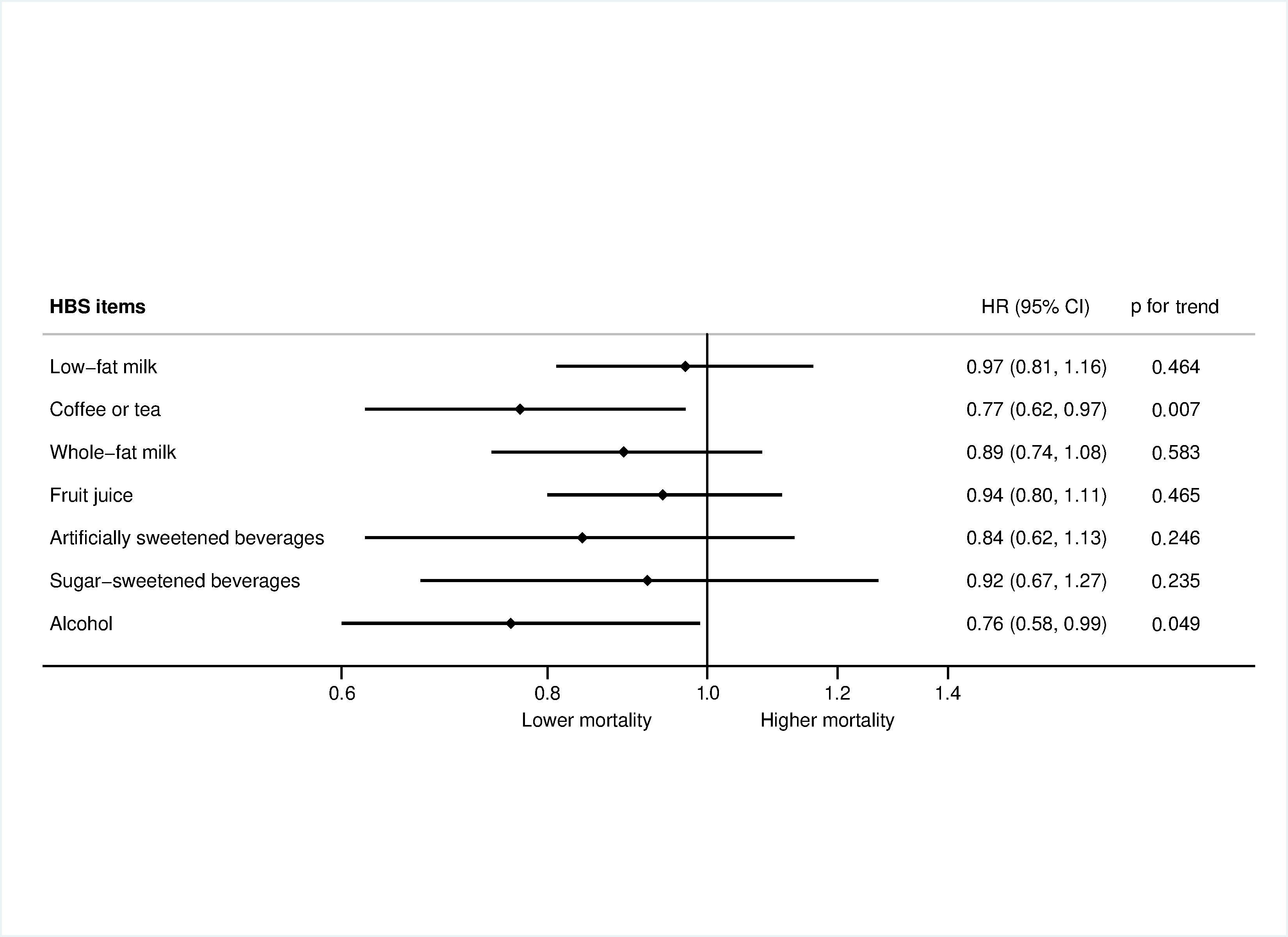

Supplement: S3 Fig — HBS, Healthy Beverage Score; HR, hazard ratio; CI, confidence interval. (TIF) [file pmed.1004337.s007.tif]
