# Supplementary material for: Association of a healthy beverage score with total mortality in the adult population of Spain: A nationwide cohort study
Source: PLoS Med. 2024 Jan 23;21(1):e1004337. doi: 10.1371/journal.pmed.1004337 (PMC10805278; doi:10.1371/journal.pmed.1004337)
Supplement: Attachment — Submitted filename: PlosMedicine - Review NOV2023.docx [file pmed.1004337.s008.docx]

November 22nd, 2023

Dear Editor of Plos Medicine:

We are truly grateful for giving us the opportunity of sending a new version of our manuscript (PMEDICINE-D-23-01967R1) now entitled: “Association of a healthy beverage score with total mortality in the adult population of Spain: A nationwide prospective cohort study”. Below, you will find a detailed answer to reviewer’s questions.

Kind regards,

The authors

We appreciate the comments made by the Academic Editor, the editors and specific reviewers, which have helped us to improve the manuscript. Below is a point-by-point response to the comments.

**Comments from the academic editor**

**I am unsure of the value because of some problematic scoring. This is not the first such study but one with a strong outcome. I do see some issues but let’s see how the authors respond.**

A: Thank you for your comment. We have addressed some of the scoring issues in the new version of the manuscript. This is the first time that a score on beverages is being assessed in the general population. A previous manuscript on total mortality was focused on a very selected sample (patients with mild to moderate chronic renal failure). On the other hand, total mortality is a hard variable in epidemiology because of its validity and reproducibility. In addition, we believe that our findings are relevant because most indices are only based on solid food, which underestimates the role of beverages in health. I hope our response will be adequate.

**Comments from the editors**

**General:**

**The editorial team are in agreement that your manuscript could offer some contribution to the field but, we have concerns regarding novelty/advance and require that you clearly define this in the relevant parts of the manuscript.**

A: Thank you very much. We have provided further explanations and we have modified the relevant parts of the manuscript.

**Your study is observational and therefore causality cannot be inferred. Throughout, please remove language that implies causality and refer to associations instead.**

A: Following the suggestions of the editor and the reviewers, we have changed the conclusion in the abstract and in the main text. Now you can see:

Abstract: “In this study, we observed that a higher adherence to the HBS was associated with a one-quarter reduction in total mortality”.

Main text: “In conclusion, in this representative study of the adult Spanish population, a higher adherence to the HBS was associated with a one-quarter decrease in total mortality. This reduction in mortality is relevant in nutritional public health prevention.”

**Please add line numbers starting at 1 on the title page and in continuous sequence throughout thereafter.**

A: Line numbers have been added.

**Please ensure that the study is reported according to the STROBE guideline, and include the completed STROBE checklist as Supporting Information. Please add the following statement, or similar, to the Methods: "This study is reported as per the Strengthening the Reporting of Observational Studies in Epidemiology (STROBE) guideline (S1 Table)."**

A: The statement has been added to the Methods section. We have added the STROBE checklist to Supporting Information. Accordingly, we have added a flow diagram (please, see S1 Fig).

**Data availability**

A: Following recommendation b), the data are freely available on request by contacting Esther López-García (esther.lopez@uam.es).

**Competing interests**

A: The authors have no competing interest, ties to industry, or financial stake in the results of the current study. A statement of no competing interests was included in the original submission.

**Title**

A: The title of the manuscript has been changed as follows:

“Association of a healthy beverage score with total mortality in the adult population of Spain: A nationwide prospective cohort study”

**Abstract
Background: Line 1 – suggest ‘relationship’.**

A: The word “relation” has been modified to “relationship” in line 1 of the abstract background.

**Abstract Methods and Findings:**

**Please ensure that all numbers presented in the abstract are present and identical to numbers presented in the main manuscript text.**

A: All numbers in the abstract as well in the main manuscript text have been checked.

**Please include the study design (observational cohort study), population and setting and, please clearly define the main outcome measures.**

A: In this regard, we have included in the abstract:

“Observational cohort study of community-dwelling adults from Spain.”

“Total mortality was ascertained by linkage to the Spanish National Death Index.”

**Please quantify the main results with 95% CIs and p values. Please report p as <0.001 and where higher the exact p values as p=0.002, for example. Please separate upper and lower bounds of 95% CIs with commas as opposed to hyphens as these can be confused with negative values.**

A: Done.

**Please include any important dependent variables that are adjusted for in the analyses.**

A: In the new version, we have added to the abstract methods: “Statistical analyses were performed with Cox models and adjusted for main confounders, including sociodemographic, lifestyle, dietary variables, and morbidity.”

**Please include the actual amounts and/or absolute risk(s) of relevant outcomes not just relative risks or correlation coefficients. (example for absolute risks: PMID: 28399126).**

A: Following the recommendation we have added the following abstract results: “corresponding to a 8.3% reduction in the absolute risk of death.”

In the main text (Results) we have added the following:

“The decrease in absolute risk of death was 4.3% for quartile 2, 6.3% for quartile 3, and 8.3% for quartile 4.”

**In the last sentence of the Abstract Methods and Findings section, please describe the main limitation(s) of the study's methodology.**

A: The main limitation of this study was added in the Abstract Methods as follows: “The observational design of the study did not allow us to draw causal conclusions.”

**Abstract Conclusions:**

**Please address the study implications without overreaching what can be concluded from the data; the phrase "In this study, we observed ..." may be useful. Please interpret the study based on the results presented in the abstract, emphasizing what is new without overstating your conclusions. Please avoid vague statements such as "these results have major implications for policy/clinical care". Mention only specific implications substantiated by the results. In revising your conclusions, please ensure to avoid assertions of primacy ("We report for the first time....")**

A: In abstract conclusions, we have added the following sentence:

“Adherence to a healthy beverage pattern could play a role in the prevention of premature mortality.”

**Author summary**

**Author summary bullet points have been added as follows:**

A: We have added the following bullet points:

• Why Was This Study Done?

- Most dietary patterns include only solid food, but beverages also play an important role in our diet.
- We described a healthy beverage dietary pattern , which includes low-fat milk, coffee and tea, whole-fat milk, fruit juice, artificially-sweetened beverages, sugar-sweetened beverages and alcohol.
- Our aim was to assess its relationship with total mortality.

• What Did the Researchers Do and Find?

- Participants were categorized according to their adherence to the healthy beverage dietary pattern.
- We found that a higher adherence to the healthy beverage score was associated with a lower all-cause mortality in the adult Spanish population.

• What Do These Findings Mean?

- Adherence to a healthy beverage pattern could serve as a potential diet-based strategy to prevent premature mortality.

**INTRODUCTION
As above, please indicate whether your study is novel and how you determined that. If there has been a systematic review of the evidence related to your study (or you have conducted one), please refer to and reference that review and indicate whether it supports the need for your study.
Please conclude the Introduction with a clear description of the study question or hypothesis as in the current version.**

A: There is no systematic review or narrative review on this topic. A sentence has been added to the introduction to address this request:

“We hypothesize that a higher adherence to a healthy beverage score could be associated with lower mortality.”

**Methods and results**

**As above, please add the following statement, or similar, to the Methods: "This study is reported as per the Strengthening the Reporting of Observational Studies in Epidemiology (STROBE) guideline (S1 Table).**"

A: Done.

**Did your study have a prospective protocol or analysis plan? Please state this (either way) early in the Methods section.**

**a) If a prospective analysis plan (from your funding proposal, IRB or other ethics committee submission, study protocol, or other planning document written before analyzing the data) was used in designing the study, please include the relevant prospectively written document with your revised manuscript as a Supporting Information file to be published alongside your study, and cite it in the Methods section. A legend for this file should be included at the end of your manuscript.
b) If no such document exists, please make sure that the Methods section transparently describes when analyses were planned, and when/why any data-driven changes to analyses took place.
c) In either case, changes in the analysis-- including those made in response to peer review comments-- should be identified as such in the Methods section of the paper, with rationale.

For all observational studies, in the manuscript text, please indicate:
(1) the specific hypotheses you intended to test,**

**(2) the analytical methods by which you planned to test them,**

**(3) the analyses you actually performed, and**

**(4) when reported analyses differ from those that were planned, transparent explanations for differences that affect the reliability of the study's results. If a reported analysis was performed based on an interesting but unanticipated pattern in the data, please be clear that the analysis was data-driven.**

A: Our analysis was planned following the publication of a previous article on this score and frailty in older adults.

There was a change in the classification of alcohol compared to the previously published article. The change was that the healthy category for alcohol was "no consumption and moderate consumption", as recommended in the general population. In the older adult population, we used only "moderate consumption" as the healthy category. This is because moderate alcohol consumption is often recommended for older people to reduce their cardiovascular risk, but is not recommended for the general population with a lower cardiovascular risk. The results are similar with both approaches.

We have added the following in the Methods section: "This was a preplanned analysis. The HBS was previously used in a sample of older adults where moderate alcohol consumption was considered the healthy option due to their high cardiovascular risk (18). However, in the general population with lower cardiovascular risk, the category of alcohol that is considered healthy is no consumption or moderate consumption.”

**As per reviewer comments (see below), please include further details of how this score differs from the HBI and the added advantage and please justify/explain your chosen scoring system/criteria.**

A: HBS varies slightly from the Healthy Beverage Index (HBI) by Duffey and Davy's (1) in the following aspects:

1) The HBS does not include water consumption as this information was not available in our dataset. Water is the recommended beverage that does not provide energy, macronutrients or micronutrients. Therefore, water consumption can be considered to be free for the general population.
2) Energy from beverages is not included in the HBS because the HBS is intended to be used as a checklist or screener in the general population. A food composition table is required to calculate total beverage energy and is not practical for use in the general population or in clinical settings. On the other hand, energy from beverages depends on beverage consumption, therefore this item is not independent from the rest of the other items.
3) The HBS does not include fluid requirements because the HBI was estimated by using a formula based on water consumption and energy from beverages. Therefore, this item is not independent from the rest of the other items.
4) Cut-off points were modified to fit with the Spanish beverage consumption.
5) In the index by Duffey and Davy’s no alcohol consumption was considered to be unhealthy. We considered that no alcohol consumption is a healthy habit.

The HBS that we used was similar to that used by EA Hu (2) except for:

1) Cut-off points were modified to fit with the Spanish beverage consumption.
2) In the score by Hu no alcohol consumption was considered to be unhealthy. We considered that no alcohol consumption is a healthy habit.

The HBS is a practical score adapted for use in the general population (similar to that used by EA Hu). Therefore, our results could be generalized and used as recommendations for the general population.

We have changed the Methods sections as follows:

“A Healthy Beverage Index (HBI) was previously described by Duffey and Davy (11) and a Healthy Beverage Score (HBS) was applied by Hu et al (15). Based on the HBS, we built the 7-item HBS modifying its cut-off points to fit with the beverage consumption obtained from this representative sample of the adult Spanish population.”

References:

(1) Duffey KJ, Davy BM. The Healthy Beverage Index Is Associated with Reduced Cardiometabolic Risk in US Adults: A Preliminary Analysis. J Acad Nutr Diet [Internet]. 2015 Oct 1;115(10):1682-1689.e2. Available from: http://www.jandonline.org/article/S2212267215005420/fulltext
(2) Hu EA, Anderson CAM, Crews DC, Mills KT, He J, Shou H, et al. A Healthy Beverage Score and Risk of Chronic Kidney Disease Progression, Incident Cardiovascular Disease, and All-Cause Mortality in the Chronic Renal Insufficiency Cohort. Curr Dev Nutr [Internet]. 2020 [cited 2022 Aug 23];4(6):nzaa088. Available from: /pmc/articles/PMC7293206/

**Please define the length of follow up (eg, in mean, SD, and range).**

A: We have added the following:

“After a mean follow-up of 12.5 years (SD: 1.7; range: 0.47-13.9) and 151,459 person-years, a total of 967 deaths occurred.”

**As for the abstract, please quantify the main results with 95% CIs and p values. Please report p as <0.001 and where higher the exact p values as p=0.002, for example. Please separate upper and lower bounds of 95% CIs with commas as opposed to hyphens as these can be confused with negative values.**

**Page 13 – ‘(95% CI, 0.55-0.88, P for linear trend=.003)’ please report as follows, in line with previous formatting requests/requirements, ‘(95% CI [0.55,0.88], p for linear trend=0.003).**

A: Done.

**Tables**

**Thank you for indicating that your analyses are adjusted and detailing the factors adjusted for. To help facilitate transparent data reporting, please also include unadjusted analyses for comparison.**

A: The Cox model we have set for the analysis has staggered entries (including the date of birthday), therefore the unadjusted model adjusts for age to a certain degree. In the new version, an unadjusted model was reported in Table 4 (model 1).

**Please ensure that all abbreviations re defined in the caption/footnote including those used for statistical reporting – CI, HR, BMI, for example. Please check and amend throughout including in the supporting files where relevant.**

A: All abbreviations have been added to the footnote of the tables. In addition, other abbreviations throughout the manuscript and supporting files have also been revised.

**Throughout, please separate upper and lower bounds of 95% CIs with commas as opposed to hyphens as the latter can be confused with the reporting of negative values.**

A: Done.

**Table 1 – the footnote appears to detail how the scoring system is applied to different beverages. This would be better presented as a table.**

**Please revise accordingly and in line with the guidance above regarding the requirement for more nuanced details of the scoring system.**

A: A supplementary table has been added indicating the cut-off points used in each item to classify participants according to its consumption and sex. Please, see supplementary table 2 (S2 Table).

**Table 2 (and others) – please report p as <0.001 as opposed to <.001. Please check and amend throughout all sections of the manuscript including text, tables, figures including in the supporting files.**

A: Done.

**Figures**

**Please ensure that all abbreviations including those used for statistical reporting are clearly defined in an appropriate caption/footnote.**

A: Abbreviations in Figure 1 were checked.

**Please ensure that p values are reported as <0.001 and where higher the exact p values as p=0.002, for example.**

A: Done.

**Please replace uppercase ‘P’ with lowercase ‘p’.**

A: Done.

**Where adjusted analyses are presented to help facilitate transparent data reporting, please also present unadjusted analyses for comparison.**

A: We have added supplementary figure 2 and supplementary figure 3 with the unadjusted figures. Please, see S2 Fig and S3 Fig.

**Where 95% CIs are reported, please also report p values as detailed above.**

A: Done.

**Discussion**

**Please present and organize the Discussion as follows: a short, clear summary of the article's findings; what the study adds to existing research and where and why the results may differ from previous research; strengths and limitations of the study; implications and next steps for research, clinical practice, and/or public policy; one-paragraph conclusion.**

A: Thank you. We think that the discussion is correctly organized.

**Please refrain from using sub-headings such that the discussion reads as continuous prose.**

A: As recommended, the sub-heading corresponding to “conclusions” has been removed.

**Please see reviewer #2 comments detailed below regarding wider discussion of the HBS which we agree with.**

A: All comments and questions from reviewer #2 have been considered and answered below.

**Page 19 – please remove the sub-heading ‘conclusions’.**

A: As recommended, this sub-heading has been removed.

**Declarations**

**Page 22 – please remove all declarations from the main manuscript and include only in the masncuript submission form. In the event of publication, these details will be compiled as metadata.**

A: All declarations have been removed from the main manuscript.

**References**

**PLOS uses the reference style outlined by the International Committee of Medical Journal Editors (ICMJE), also referred to as the “Vancouver” style.
Please see our website for further guidelines here https://journals.plos.org/plosmedicine/s/submission-guidelines#loc-references
Journal name abbreviations should be those found in the National Center for Biotechnology Information (NCBI) databases.**

**Please ensure that all web references include an ‘Accessed [date]’ as opposed to cited. Journal publications accessed on-line should not be cited as web references but as journal publications using the format detailed above.**

A: All references have been revised as recommended.

**Supporting information**

**As above, please include the completed STROBE checklist as Supporting Information. When completing the checklist please refer to section and paragraph numbers as opposed to page or line numbers as these often change in the event of publication.**

A: The completed STROBE checklist has been added as Supporting Information (S1 Table). Accordingly, we have added a flow diagram (please, see S1 Fig).

**S1 table – as for the tables in the main manuscript, please separate upper and lower CI bounds with commas as opposed to hyphens. Please replace ‘P’ with ‘p’ in the column header. Please report p <0.001 and where higher the exact p value as p=0.002, for example. Please ensure to amend the footnote also. To help facilitate transparent data reporting, please also present unadjusted analyses for comparison.**

A: Recommendations regarding CI bounds, lowercase “p” and p value have been checked and modified. The label of this table was changed to S3 Table. Also, unadjusted analyses have been added.

**Comments from the reviewers:**

**Reviewer #1: Alex McConnachie, Statistical Review**

**The paper by Rodríguez-Ayala looks at the association between a derived healthy drinking score and mortality, using a large cohort study from Spain. This review considers the use of statistics in the paper.**

**Overall, the analysis is very good. The use of Cox PH models is appropriate, and it is nice to not have to remind the authors to check the PH assumption. Models with different levels of adjustment for confounders are reported. the exposure of interest is analysed both in categories but also as a continuous measure using cubic splines. A landmark analysis is done to check for reverse causality. The individual components of the main exposure variable are also assessed. Everything is explained well and presented clearly. My comments are fairly minor.**

A: Thank you for providing us such valuable feedback. Here, you may find a point-to-point answer to your remarks.

**As ever, care should be taken to avoid language that implies a causal association, such as the use of the words "resulted in", as seen in the conclusion of the abstract.**

A: As recommended, the words “resulted in” has been modified in the conclusion of the abstract:

“In this study, we observed that a higher adherence to the HBS was associated with a one-quarter reduction in total mortality.”

No other language expressions that implies a causal association have been identified.

**Missing data are imputed using stochastic regression. What is "stochastic regression"? The reference is a book that I could not access. However, looking online, it appears to be another term for multiple imputation - if so, then this would be a more widely used term.**

A: To improve the readability of the manuscript, we have changed "stochastic regression" to "multiple regression" in the Methods section because both methods are based on the same basic procedure as explained in the referenced book:

“Independent variables with missing values were imputed by using multiple imputation [17]”.

After checking the accessibility of the book citation provided, we have considered keeping it in the manuscript.

The referenced book is available on Google Books (link: <https://books.google.com/books?id=MN8ruJd2tvgC&printsec=frontcover&dq=Enders+C.+Applied+Missing+Data&hl=es&newbks=1&newbks_redir=1&sa=X&ved=2ahUKEwj19vat-ZmCAxUCUaQEHdXlB2UQ6AF6BAgKEAI>). A free version of this book is also available at <http://hsta559s12.pbworks.com/w/file/fetch/52112520/enders.applied>.

**One of the results in Table 4 looked odd. For younger ages, the trend is nearly significant at p<0.05, with an increasing trend; for older ages, the trend is clearly decreasing. For Q3 and Q4, the confidence intervals are actually non-overlapping. Yet the interaction p-value is 0.65, which is a surprise. Could there be an error somewhere?**

A: We have done the analysis again because we detected a mistake with the alcohol. You can see results in the new table below and the CIs are now overlapping. Although we see the tendency you describe, the p value was far from being statistically significant possible due to several reasons: we used the Wald test that is not powerful in detecting interactions and the number of deaths among young people are limited (as expected) and only based on 37 deaths in the last quartile. We have checked the sintaxis again and we think there are no further errors.

| **Total mortality** | **Quartile 1**  **HR (95% CI)**  **(Less healthy)** | **Quartile 2**  **HR (95% CI)** | **Quartile 3**  **HR (95% CI)** | **Quartile 4**  **HR (95% CI)**  **(Healthier)** | **p for interaction** |
| --- | --- | --- | --- | --- | --- |
| **Age** |  |  |  |  |  |
| **≤ 60 years** |  |  |  |  | 0.672 |
| Deaths, n | 27/2433 | 46/2348 | 36/2002 | 55/2278 |  |
| Model 3 | 1 (ref.) | 1.18 [0.67,2.07] | 1.24 [0.71,2.19] | 1.35 [0.80,2.28] |  |
| **> 60 years** |  |  |  |  |  |
| Deaths, n | 114/380 | 181/637 | 192/743 | 316/1340 |  |
| Model 3 | 1 (ref.) | 0.73 [0.55,0.97] | 0.72 [0.54,0.97] | 0.64 [0.49,0.83] |  |

As suggested by reviewer 2, we have made new categories with the cut-off point at 65 years old because it makes more sense from a social point of view. We don’t see the above mentioned tendency. Now the number of deaths in quartile 4 is 79 among those younger than 65.

**The interaction p-values come from Wald tests, but I would normally use likelihood ratio tests in this situation. Do these give the same results?**

A: Thank you for this question.

Likelihood ratio (LR) tests were not calculated to check for interactions in this study. As explained in Methods subsection, Statistical analysis, the survey command was used to account for the complex sampling design. LR tests are not allowed to be used with the survey command in Stata. The reason why LR tests are not suitable with survey data is because the maximum likelihood assumption that the observations are independent would be otherwise violated. In consonance, Stata suggests the use of the *test* command (Wald test) which computes an adjusted Wald test. More information on this issue is available at:

-<https://www.stata.com/support/faqs/statistics/likelihood-ratio-test/>

-<https://www.stata.com/statalist/archive/2008-02/msg00866.html>

**There was a strange sentence in the discussion. "First, the number of deaths was low to evaluate cause specific mortality in subgroups of participants and sensitivity analyses were only performed with total mortality." As far as I can see, no analyses were done on cause specific mortality - everything was done with total mortality.**

A: The reviewer is right on this comment, everything was done with total mortality. We have removed that sentence.

**In Figure 1, what is the right-hand axis showing?**

A: Thank you for this remark. A label for the right-hand axis was added “Percentage (%)”.

**Reviewer #2:**

**The authors explore the association of a Healthy Beverage Score with mortality risk in the ENRICA Study. Beverage consumption is an important domain of dietary intake that is often left out (with the exception of alcohol intake) of dietary guidance. Overall, the manuscript is well-written, and the study is of potential public health interest; however, there are some major concerns that should be addressed.**

A: Thank you, we greatly appreciate all your comments. In the following lines, you can find a point-to-point answer to your questions.

*** As indicated in the text, the ENRICA study was drawn from a stratified cluster sample. Are the estimates provided weighted to be nationally representative? If so, please describe the sample weights and how they were applied. If not, was the enrolled sample in fact representative? Additional information on response rate is also needed.**

A: Thank you for this comment. The ENRICA study is a representative sample of the Spanish adults population. The published article on the rationale and methods of the Study on Nutrition and Cardiovascular Risk in Spain (ENRICA) (link: <https://www.sciencedirect.com/science/article/pii/S1885585711004312>) provides great detail on the study sampling. This article is also referenced in the manuscript.

The study sample comprised noninstitutionalized adults (aged 18 and older) from Spain. The participants were selected by stratified cluster sampling. In a first step, the sample was stratified by province (including the 50 provinces of Spain) and the size of the municipality (10 000; 10 000-100 000; 100 000-500 000; >500 000 population). Afterwards, the clusters were selected randomly in two stages: municipalities and census sections. Finally, the households within each section were selected by random telephone dialing using the directory of telephone land-lines as the sampling frame. Subjects in the households were selected proportionally to the distribution of the population of Spain by sex and age group (18-29, 30-44, 45-64, ≥ 65 years). Only 1 person was selected in each household. In the telephone call, the overall objectives and procedures of the study were explained; a formal letter of invitation and detailed written information on the study were sent to the participant’s home. Several days later, a second telephone call was made to confirm the participation. Information was obtained from a total of 248 municipalities and 1,241 census sections in Spain.

In this study, the response rate was 51 %. This response rate is considered to be among the highest of the National Health Interview and Examination Surveys conducted in Europe when urine and blood samples are mandatory to participate. Reasons for refusal to participate included the refusal of a blood sample extraction (51.7%) and not being interested in the study (37.8%). On the other hand, the similar sociodemographic distribution between the study participants and the Spanish population in 2009 suggests that the ENRICA study is representative of the population of Spain.

We have added the following lines in the Methods section (Study design and participants):

“Sample weights were based on the size of municipalities, sex, and age.”

“The response rate was 51% and the main reasons for non-participation was refusal to provide a blood sample (51.7%) and not being interested in the study (37.8%).”

*** The text suggests that the HBS varies slightly from Duffey and Davy's Healthy Beverage Index HBI; however, the HBI is a 10-item index that includes water, total beverage energy, and fluid requirements in addition to the 7 items included in the current HBS. Please comment on these differences and how they likely impacted the scores and results.**

A: HBS varies slightly from the Healthy Beverage Index (HBI) by Duffey and Davy's (1) in the following aspects:

1) The HBS does not include water consumption as this information was not available in our dataset. Water is the recommended beverage that does not provide energy, macronutrients or micronutrients. Therefore, water consumption can be considered to be free for the general population.
2) Energy from beverages is not included in the HBS because the HBS is intended to be used as a checklist or screener in the general population. A food composition table is required to calculate total beverage energy and is not practical for use in the general population or in clinical settings. On the other hand, energy from beverages depends on beverage consumption, therefore this item is not independent from the rest of the other items.
3) The HBS does not include fluid requirements because the HBI was estimated by using a formula based on water consumption and energy from beverages. Therefore, this item is not independent from the rest of the other items.
4) Cut-off points were modified to fit with the Spanish beverage consumption.
5) In the index by Duffey and Davy’s no alcohol consumption was considered to be unhealthy. We considered that no alcohol consumption is a healthy habit.

The HBS that we used was similar to that used by EA Hu (1) except for:

3) Cut-off points were modified to fit with the Spanish beverage consumption.
4) In the score by Hu no alcohol consumption was considered to be unhealthy. We considered that no alcohol consumption is a healthy habit.

The HBS is a practical score adapted for use in the general population (similar to that used by EA Hu). Therefore, our results could be generalized and used as recommendations for the general population.

We have changed the Methods sections as follows:

“A Healthy Beverage Index (HBI) was previously described by Duffey and Davy (2) and a Healthy Beverage Score (HBS) was applied by Hu et al (3). Based on the HBS, we built the 7-item HBS modifying its cut-off points to fit with the beverage consumption obtained from this representative sample of the adult Spanish population.”

We have added in the limitation section:

“Third, water consumption was not available in this study. Water is universally recommended as a safe beverage and as the main source of hydration. As water does not provide energy, macronutrients or micronutrients, its consumption is considered free for the general population."

*** What is the rationale behind 4-point scoring system for components? It is unclear why alcohol, fruit juice, and artificially sweetened beverages are only assigned values 1 and 4. Scoring these components as either 1 or 4 appears to have resulted in some scores being more probable than others such that Q4 has almost 900 more individuals than Q3.**

A: Thank you for this comment. Different aspects are to be considered:

1) Items on fruit juice, artificially-sweetened beverages scored 1 to 4 to ensure that all items have the same weight in the final index. This is a common way to design “a priori” dietary patterns. The lack of consumption of these beverages in most of the participants did not allow us to make categories.

2) Regarding alcohol, there is not a linear association between alcohol and mortality. Our score simply penalizes excessive alcohol consumption. In literature, there is a general agreement on this point when studying the general population.

3) Differences in sample size for Q3 and Q4 are due to the specific distribution of the index in our sample.

**Additionally, according to the footnote for table 2, the quartiles are very similar for men and women. If this is correct, what is reason for sex-specific quartiles?**

A: Thank you for this question. You are right with this comment. However, in nutritional epidemiology there is a long tradition to study sex-specific quartiles because the amount of food consumed depends substantially on body size (very different between sexes). We think they should remain this way, although the results would potentially not change.

*** The alcohol consumption cutoff for women does not include women who drink 20-23 g/day. As it stands, it is <20 g/day for a score of 4 and >=24 g/day for a score of 1. Please redefine/clarify the alcohol consumption cutoff for women.**

A: Thank you for this remark. An amend regarding the alcohol consumption cut-off for women has been made in the text and in the footnote of Table 1 as it corresponds to < 24 g/day for moderate consumption:

“Finally, for alcohol consumption, participants with no consumption or moderate drinkers (< 40 g/day for men and < 24 g/day for women) scored 4, and heavy drinkers (≥ 40 g/day for men and ≥ 24 g/day for women) scored 1.”

^“b^ Heavy drinkers were defined as consumption ≥ 40 g/day for men and ≥ 24 g/day for women. Among drinkers, a moderate alcohol consumption was defined as < 40 g/day for men and < 24 g/day for women.”

When reviewing the statistical analyses, we also found a mistake concerning alcohol categorization, which has been corrected in the current version. This mistake affected the score and the number of deaths in Q3 and Q4. We have corrected this mistake and the results and conclusions are similar.

**Also, alcohol consumption (table 2) seems quite low in comparison to other beverage consumption. How is alcohol intake defined, as ethanol or volume of alcoholic beverages? Were there a large proportion of zeros (i.e., nondrinkers) for certain beverages? If so, it would be more informative to show the median and IQR rather than mean (SD).**

A: Alcohol intake was considered as ethanol consumption in grams per day to take into account the differences in alcohol content between wine, beer, and spirits. A total of 43.6% of the sample are nondrinkers (mainly women). In the new Table 3, we have maintained alcohol consumption as a mean and standard deviation. The median and IQR for alcohol consumption in Quartile 1 was 2.9 (0.0,14.6), Quartile 2 was 2.9 (0.01, 14.2), Quartile 3 was 0.94 (0.0,11.1) and 0.09 (0.0,8.6) (p<0.001).

*** It is unclear what the footnote under table 1 is specifying. Are these the scores of the participants within those tertiles and quartiles?**

A: To clarify this we have added a supplementary table (S2 Table) with the cutt-off point for each item according to sex.

We have added to Results section:

“Sex-specific cut-off points for individual items of the HBS are shown in S2 Table.”

*** The cutoff values for the interaction tests appear to be designed to preserve sample size in strata but do not necessarily make sense if a biological interaction is suspected. The authors should consider more clinically meaningful categories or, if statistical power is the main concern, run models with continuous variables and their interactions.**

A: We appreciate this comment. We have tried to make more clinically meaningful categories. We have changed the categories for age (now the cut-off point is 65 years, the age for retirement in Spain) and BMI (obesity: ≥30 Kg/m^2^).

*** Given the wide age range of the population, age-standardized descriptive statistics for table 2 would be more helpful in understanding what other covariates are potential confounders. For this reason (and given the strong potential of age to confound the HBS-mortality association), age should also be considered as the underlying time metric.**

A: Thank you for this comment. Table 2 is now age-adjusted. Also, as the reviewer suggested, we have recalculated the analyses considering staggered entries using age as the underlying time metric (Table 4).

Also, we have changed the description of statistical analysis in this regard:

“Across sex-specific quartiles of adherence to the HBS, age-adjusted baseline characteristics of participants were computed using marginals. Age-adjusted means and standard errors (SE) for continuous variables and age-adjusted proportions (%) for categorical variables were provided. To estimate Hazard Ratios (HR) and their 95% Confidence Intervals (CI), Cox proportional hazards regression models were built and age was considered as the underlying time metric. The survey command was applied to account for the complex sampling design. The lowest category of adherence to the HBS was used as reference.”

The results obtained were similar, but a little bit more conservative.

*** Presently, one would expect multivariable adjustment for these variables to alter HR estimates; however, in Table 3, adjustment does not meaningfully shift estimates. This is surprising, given that coffee consumption, which tends to be highly correlated with smoking, is highlighted as explaining most of the association.**

A: Thank you for this comment. Age and sex are the variables that potentially affect the most the results. Age has been partially adjusted by using the age as the underlying time metric, and sex making the quartiles sex-specific. Apart from that, the number of variables in the model is high and subsequently results are multi-dimensional. It is noteworthy that models 2 and 3 are similar, both adjusting for diet, but with different approaches. However, we have decided to include in the model the status of ex-drinker (as a marker for morbidity) that makes the results a little bit more conservative.

*** Table 4 - the number of deaths for each quartile do not equal the number of deaths provided in table 3. If correct, please explain why the cohort is different between the full analysis and stratified analyses.**

A: Thank you for this comment. This is due to a mistake in the sintaxis dealing with item 7 (alcohol). The mistake has been corrected in Table 4, since the number label has changed. Results were similar.

*** Table S1: Why are there more deaths now in Quartile 1 and Quartile 3 than in the original analysis? Were the quartiles redefined for the sensitivity analysis?**

A: In this analysis, deaths during the three first years of follow-up were not considered and the number of deaths were different. The label of this supplementary table was changed to S3 Table.

*** There is no discussion on the actual HBS scale itself, only on its component beverages. The novelty of this study is the use of a HBS score, and advantages, implications, and limitations of the score should be more directly discussed.**

A: Thank you for this remark. The aim of our study was to provide an easy, accessible tool to make recommendations about beverage consumption in the clinical setting. Items such as total beverage energy and fluid requirements would not be suitable for application in clinical practice. Along with other well-known nutritional recommendations for the consumption of good quality solid foods, recommendations on most commonly consumed drinks by the general population could be useful for the physicians as they will be able to provide indications on good quality beverage consumption as well.

In the Discussion section, we have added:

“The use of the HBS as an overall measure of beverage consumption has several advantages. First, the use of this score overcomes the limitations of analyses of relationships between individual beverages and diseases, as beverage consumption may be correlated, and an increase in consumption of one beverage may be associated with a decrease in the others. Secondly, the HBS could be a complementary tool for assessing adherence to dietary patterns that include only solid foods, in order to assess dietary quality as a whole. Thus, the HBS could serve as a simple and rapid screener to obtain information on the quality of beverage consumption from the general population, similar to other indexes used to assess adherence to certain diets, such as the Mediterranean diet. Also, the use of this 7-item pattern may be an optimal choice when dealing with patients in time-constrained clinical settings. Finally, the HBS includes items on commonly consumed beverages and could be easily adapted to other populations with only minor modifications to account for their specific beverage consumption.

We have used the HBS in the general population and caution should be exercised in deriving beverage consumption recommendations from this score in specific populations, especially those with restricted fluid requirements, long-term liquid diets, and other pre-existing conditions involving fluid consumption. Further studies are also needed in specific population subgroups. In addition, a future study could consider intercorrelations and specific population-based patterns of beverage consumption using an “a posteriori” approach.”

**Minor comment:**

*** Visually inspecting the Schoenfeld residuals is not a statistical test for the proportional hazards assumption. Please replace the word "test" with "assess".**

A: Done.

**Reviewer #3:**

**The article proposes the evaluation of the association of the adherence to a Healthy Beverage Score (HBS) and all-cause mortality in the ENRICA-1 cohort, a representative sample of the adult Spanish population. The article concerns an argument of current interest and is in general clear and well-written; the data come from a well-designed and well-conducted study with updated references. Few points should be addressed before considering for publication.**

A: Thank you very much for your comments and questions. In the following lines, we answer each one of them.

**1. In Figure 2, each item should be adjusted by the rest of the items that are part of the index (each beverage by the rest of the beverages).**

A: Done. As recommended, a new figure 2 has been adjusted for the rest of the items that are part of the index.

In the Methods section, the following has been added:

“Also, individual items of the HBS were assessed according to model 3 plus adjustment for the remaining items that are part of the score.”

In the Results section, the following has been added:

“When individual HBS items were analyzed using Model 3 for adjustment, a higher consumption of coffee and tea, and no consumption of fruit juices and artificially sweetened beverages contributed most to the association with lower all-cause mortality (Fig 2). Unadjusted results are also shown (S3 Fig).”

**2. Sensitivity analyses could be done for chronically ill patients to test if HBS also predicts mortality among the sickest.**

A: Thank you. A new sensitivity analysis for chronically ill patients was performed and added to Table 5. As you can see in Table 5 a healthy HBS is associated with a lower mortality among those having at least one chronic condition. This finding is in line with a previous paper in which a higher adherence to the HBS was associated with a lower incidence of frailty among older adults. We have added in the result section:

“No significant interactions were found for age, sex, BMI, physical activity, vegetable consumption or adherence to the Mediterranean diet (without including alcohol). However, a statistically significant interaction was found when stratifying for the presence of at least one chronic condition (p=0.030) with lower mortality among those who were chronically ill.”

In the Discussion section, we have added:

“For those with higher adherence, the risk of death was reduced by a quarter. The association was linear and robust. It may also be particularly relevant in people with pre-existing conditions.”

**3. Another sensitivity analysis would be to evaluate if the results are modified among those who have a low consumption of vegetables.**

A: A new sensitivity analysis for vegetable consumption was performed and added to Table 5. No interaction was identified.
