# Supplementary material for: Association of a healthy beverage score with total mortality in the adult population of Spain: A nationwide cohort study
Source: PLoS Med. 2024 Jan 23;21(1):e1004337. doi: 10.1371/journal.pmed.1004337 (PMC10805278; doi:10.1371/journal.pmed.1004337)
Supplement: Attachment — Submitted filename: R2 - Plos Medicine - Dic 2023.docx [file pmed.1004337.s009.docx]

December 21 st, 2023

Dear Editor of Plos Medicine:

We are grateful for giving us the opportunity of resubmitting the revised version of our manuscript (PMEDICINE-D-23-01967R2) now entitled: “Healthy beverage consumption and total mortality in Spanish adults: A nationwide cohort study”. Below, you will find a detailed answer to reviewer’s questions.

Kind regards,

The authors

Once again, we are grateful for the comments made by the Academic Editor, the Editors and specific reviewers. Below is a point-by-point response to all your questions and comments.

**COMMENTS FROM THE ACADEMIC EDITOR**

**I think the authors have taken their data as far as they can. Their measure is really quite crude but pending the reviews, I am reluctantly ok with this. The authors really must be cautious in their findings. This points toward an impact but more refined measures are needed to totally test this relationship.**

A: Thank you for your comments. In the new version of the manuscript we have interpreted the results more cautiously and toned down the conclusions.

**COMMENTS FROM THE EDITORS**

**GENERAL**

**Thank you for detailed responses to previous editor and reviewer comments.**

**The Editorial team agree with the Academic Editor that you need to be cautious about how you report your findings. Specifically, we require greater consideration and contextualization of actual versus relative risk when reporting and discussing your results. Please see below for further comments which we require you address in full prior to publication.**

A: Thank you. We have toned down the reporting of results and conclusions to take into account absolute versus relative risk.

**TITLE**

**Please revise your title, specifically description as ‘prospective’ is misleading. Suggest the following, “Healthy beverage consumption and total mortality in Spanish adults: A nationwide cohort study”.**

A: Thank you. The title has been changed in accordance with your suggestion.

**DATA AVAILABILITY STATEMENT**

**Thank you for updating your statement in line with point ‘b)’ below. Your statement requires revision. Please also indicate where these data are held i.e., where does the contact for data requests reside?**

**‘b) If the data are owned by a third party but freely available upon request, please note this and state the owner of the data set and contact information for data requests (web or email address). Note that a study author cannot be the contact person for the data.’**

A: We have added the following contact information as suggested:

The data are freely available upon request by contacting Esther López-García at the Department of Preventive Medicine and Public Health, Faculty of Medicine, Universidad Autónoma de Madrid-IdiPaz, CIBERESP (CIBER of Epidemiology and Public Health), 28029, Madrid, Spain. E-mail address: [esther.lopez@uam.es](mailto:esther.lopez@uam.es).

The owner of the data is the Universidad Autónoma de Madrid. The data will be hosted on a server of our institution, the Universidad Autónoma de Madrid. We have already started the procedures for its hosting.

**ABSTRACT**

**Line 29 – suggest removing ‘…the ENRICA-1 cohort…’**

A: Thank you. We have amended this sentence as follows:

“The aim of this study was to assess the association of the adherence to a Healthy Beverage Score (HBS) and all-cause mortality in a representative sample of the Spanish adult population.”

**Line 31 – suggest making reference to the ENRICA-1 cohort here instead. Perhaps, ‘We conducted an observational cohort study leveraging data from the ENRICA-1 cohort, a study of community dwelling adults in Spain set up to…[please provide brief details]…’ .**

A: As suggested, this sentence was modified:

“We conducted an observational cohort study using data from the Study on Nutrition and Cardiovascular Risk in Spain (ENRICA), which included 12,161 community dwelling individuals aged ≥18 years recruited in 2008-2010 and followed until January 2022.”

**Please also define the acronym, ‘ENRICA-1’ at first use.**

A: Done. As explained later and to avoid misinterpretations, ENRICA instead of ENRICA-1 is used to name the study.

**Line 38 – suggest moving sentence beginning ‘The HBS ranges from…’ to line 35 following ‘…(highest adherence)’.**

A: Done. The sentence in line 38 was moved to line 35:

“The HBS consists of seven items, each of which is scored from 1 to 4 (highest adherence). The HBS ranges from 7 to 28 points with a higher score representing a healthier pattern.”

**Line 45 – please place CIs in brackets as follows, ‘[0.57, 0.91]’.**

A: Done.

“For all-cause mortality, the fully adjusted Hazard Ratio for the highest vs. lowest sex-specific quartiles of HBS was 0.72 (95% Confidence Interval [0.57,0.91], p linear-trend=0.015), corresponding to an 8.3% reduction in the absolute risk of death.”

**Line 48 – this statement is true of all observational studies. Please detail the limitations of the methodology as specific to your study.**

A: Thank you for this remark. We have removed that sentence from the abstract and we have added the following:

“The main limitation was that repeated measurements on beverage consumption were not available and beverage consumption could have changed during follow-up.”

**Line 51 – ‘…associated with a one-quarter reduction in total mortality.’ This is an overstatement of your findings. Please revise and temper this statement (and later in your discussion, please see below).**

A: We have temper down the conclusion:

“In this study, we observed that higher adherence to the HBS was associated with lower total mortality.”

**As above, it is vital that risk is contextualized appropriately in relation to actual and relative measures. While the reported percentage change is correct in proportional terms it does not reflect the true change in risk. Please amend (and throughout) this is a prerequisite to publication.**

A: We understand this statement and have temper the conclusions of the study.

**AUTHOR SUMMARY**

**Thank you for including an author summary. As written, it is lacking sufficient detail. Please revise.**

**Line 74 – this would be better placed under the section at line 79 when detailing what you did.**

A: The sentence in line 74 has been moved to line 79. We have modified the author summary to be better organized and more informative.

**Please also revise this statement to improve clarity. Would it not be better to refer to the healthy beverage score (for consistency with the rest of the manuscript)?  Suggest, ‘We describe a healthy beverage dietary score based on patterns of consumption of low-fat milk, coffee and tea, whole-fat milk, fruit juice, artificially-sweetened beverages, sugar-sweetened beverages and alcohol.’ Or similar**

**Line 79 – what did the researchers do and find? This section is very vague. It is impossible to understand how you conducted your study in whom or in how many when reading this section. Please revise including [in brief] the critical details.**

A: Thank you. This section has been revised accordingly:

“Author summary:

- Most dietary patterns focus solely on solid foods, and the role of beverages as a whole has received little attention.
- Our aim was to assess the relationship between a Healthy Beverage Score (HBS) and mortality in a representative sample of community-dwelling individuals from Spain.
- Our hypothesis was that high adherence to the HBS would be associated with lower mortality.

What Did the Researchers Do and Find?

- We included a representative sample of 12,161 adults (18 and over) from Spain who were recruited in 2008-2010 and followed up until 2022. A total of 967 deaths occurred.
- Participants were categorized according to their adherence to the HBS.
- A higher total score was achieved with a higher consumption of low-fat milk, and coffee and tea, no consumption of whole-fat milk, fruit juice, artificially sweetened beverages, sugar-sweetened beverages, and no consumption or moderate consumption of alcohol.
- Each HBS item scored from 1 (minimum adherence) to 4 points (maximum adherence) and the HBS ranged from 7 to 28 points. The higher the HBS, the healthier.
- When comparing extreme categories, higher adherence to the HBS was associated with lower all-cause mortality in the Spanish adult population, with an 8.3% reduction in the absolute risk of death.

What Do These Findings Mean?

- The adherence to the HBS could serve as a potential diet-based strategy to prevent premature mortality.
- The quality of beverage patterns could influence health outcomes in the general population.”

**INTRODUCTION**

**Final paragraph – it might be helpful to indicate that you develop a modified HBS (per methods section, line 158 onwards) as part of your study.**

A: Thank you. We have added the following sentence:

“We hypothesized that a higher adherence to a 7-item Healthy Beverage Score (HBS), as previously proposed by Hu et. al. (12) and adapted to the Spanish beverage consumption, could be associated with lower mortality.”

**METHODS and RESULTS**

**Please see reviewer comments below regarding the description (and discussion later) of the HBI and HBS which we agree with. Please revise accordingly.**

A: We have revised and changed the description of the HBS accordingly.

**Throughout, please ensure that you do not overstate your findings. Please ensure adequate contextualization of the actual Vs relative risk. As reported the true risk could be over interpreted.**

A: In order not to over interpret the results and conclusions we have removed from the entire manuscript the reference to a one-quarter reduction in mortality, indicating only that the adherence to the HBS was associated with lower mortality, emphasizing the absolute value over the relative value.

We have modified:

1. The conclusion in the abstract. “In this study, we observed that a higher adherence to the HBS was associated with lower total mortality.
2. The first paragraph of the discussion: “Those with higher adherence to the HBS had an 8.3% reduction in absolute risk of death compared to those with lower adherence.”
3. The conclusion in the main text. “In conclusion, in this representative study of the Spanish adult population, higher adherence to the HBS was associated with lower total mortality”.

**Line 140 - Ethics statement – can you please detail that this statement regarding consent applies to entry into the ENRICA-2 cohort study, as we understand things. Apologies if I have misunderstood but perhaps all the more reason to clarify!**

A: Our analyses are conducted with data from the ENRICA Study that is a representative sample from the whole country (18 years old and over) conducted in 2008-10. We prefer to maintain this name (just ENRICA) in the whole manuscript.

Years later, we also followed senior participants from the original ENRICA Study, which constituted the Seniors ENRICA-1 cohort. In 2017, we started another cohort, the Seniors ENRICA-2 study with another sample only based on a region of Spain (Madrid Community). Data from Seniors ENRICA-2 were not used. We kept the acronym because the methods used for data collection were similar in the 3 studies (ENRICA, Seniors ENRICA-1, Seniors ENRICA-2).

In the Methods section, we have modified: “All participants from the ENRICA Study gave written informed consent after explaining the details of the study”.

**TABLES**

**Throughout please replace ‘Men’ and ‘Women’ with ‘Male’ and ‘Female’ respectively. Please also ensure to amend footnotes/caption where relevant.**

A: We have revised all the tables and modified them as suggested.

**Please also see statistical reviewer comments below regarding table 2, which we agree with.**

A: Thank you. We have responded to this below, in the statistical reviewer's comments.

**FIGURES**

**Figure 2 (forest plot) – please revise the presentation of p values which should be reported as <0.001 and where higher the exact p value as 0.442, for example. Please revise.**

A: Done.

**DISCUSSION**

**Please include a more nuanced discussion of the HBI Vs HBS as per reviewer comments below.**

A: Please, below, see the answer to the reviewer 2.

**Line 376 – ‘In this large prospective population-based study…’ as for the title, please remove the word ‘prospective’, whilst the data from the ENRICA-1 cohort were collected prospectively, the data were analyzed retrospectively and as such your study cannot be described as a prospective study.**

A: Done.

**Line 378 – ‘the risk of death was reduced by a quarter’. As above please temper this statement and contextualize your findings in terms of actual risk – not just proportional change.**

A: All the manuscript has been tempered as suggested.

**Line 380 – it be helpful to further quantify this statement based on the data generated.**

A: Thank you for this remark. The sentence in line 380 was modified, also maintaining the temper in the results, as recommended:

“It may also be of particular interest to people with pre-existing chronic conditions as they had lower mortality, although these findings need to be confirmed in future research.”

**Line 499 – please remove the word ‘remarkable’.**

A: Done.

**Line 501 – the phrase ‘on the other hand’ is in appropriately placed here. Please remove and perhaps replace with ‘in addition’, or similar.**

A: Thank you. We have replaced the sentence as follows:

“In addition, we used a dietary history that allowed us to collect information on beverages with validity and reproducibility in a Spanish population.”

**Line 502 – what are the ‘vital statistics records’ you refer to here? This phrase has not been used elsewhere in the manuscript. Please revie and be in explicit.**

A: Thank you for this remark. The sentence in line 502 was changed:

“Also, the national vital statistics records, accessed through linkage to the Spanish National Death Index, ensured an extensive follow-up of the cohort for mortality assessment.”

**Line 506 – as above the statement, ‘one quarter reduction in total mortality’ is an over interpretation and could be misleading, please revise.**

A: This statement has been changed as explained above.

**SUPPORTING INFORMATION**

**Thank you for indicating that you analyses were preplanned. Did you document your analysis plan as part of funding proposal, for example? If so, please include a copy of the statistical analysis plan as supporting information.**

A: Our idea was to perform the same analysis as in the previous published paper by Dominguez et. al (1). As required, we have added a sentence in this regard in the Methods section:

“This was a preplanned study and data analysis was conducted according to a prespecified plan (S1 Text). The HBS was previously used to assess the association between adherence to HBS and age-related frailty in a sample of Spanish older adults [19]. A minor modification was introduced in alcohol consumption classification. For older adults, moderate alcohol consumption was considered the healthy option due to their high cardiovascular risk [19]. However, in the current analysis, which involves the adult general population (aged ≥18 years) with lower cardiovascular risk, the category of alcohol considered healthy was "no consumption or moderate consumption.”

We have included a copy of the statistical analysis plan as supporting information (S1 Text).

1. Dominguez LJ, Donat-Vargas C, Banegas JR, Barbagallo M, Rodríguez-Artalejo F, Guallar-Castillón P. Adherence to a Healthy Beverage Score Is Associated with Lower Frailty Risk in Older Adults. Nutrients [Internet]. 2022 Sep 1 [cited 2023 Nov 19];14(18):3861. Available from: /pmc/articles/PMC9501204/

**Figures: S3 Figure (forest plot) – as for the main manuscript, please revise the presentation of p values which should be reported as <0.001 and where higher the exact p value as 0.442, for example.**

A: Done.

**Tables: Throughout please replace ‘Men’ and ‘Women’ with ‘Male’ and ‘Female’ respectively. For example, S2 table. Please also ensure to amend footnotes/caption where relevant.**

A: We have revised all the supporting tables and modified them as suggested.

**SOCIAL MEDIA**

**To help us extend the reach of your research, please detail any X (formerly Twitter) handles you wish to be included when we tweet this paper (including your own, your coauthors’, your institution, funder, or lab) in the manuscript submission form when you re-submit the manuscript.**

A: X addresses have been added in the submission form.

@UAM_Madrid

@CIBER_ESP

@ISGLOBALorg

@karolinskainst

@IISAragon

@CIBER_CV

[@ImdeaAlimenta](https://twitter.com/ImdeaAlimenta)

@HospitalULaPaz

@msrodrigueza10

**COMMENTS FROM THE REVIEWERS:**

**Reviewer #1: Alex McConnachie, Statistical Review**

**I thank the authors for their consideration of my previous comments. I am satisfied with their responses. My remaining observations are very minor.**

A: Once again, we appreciate your valuable feedback. Here, you may find a point-to-point answer to your remarks.

**I note that Table 2 has been changed. It now shows standard errors for the mean of the continuous measures. I am not keen on this, since the aim of this table should be to show the distribution of the data, whereas the SE is a measure of precision. Could these SE's be replaced with standard deviations, or simply omitted, showing the mean only?**

A: SE haven been omitted.

**Incidentally, the values reported as the SE for mean age do not look right - they seem far too large.**

A: For age, the SD was reported instead of the SE. We are sorry for the mistake.

**There is an oddly phrased sentence on lines 308-309: "…with lower mortality among those who were chronically ill" - I believe something like "with higher HBS associated with lower mortality among those who were chronically ill, but no association in those without a chronic illness" would be more accurate.**

A: Thank you. The sentence in lines 308-309 was revised and modified as suggested:

“However, a statistically significant interaction was found when stratifying for the presence of at least one chronic condition (p=0.030). Among those with at least one chronic condition, higher adherence to the HBS was associated with lower mortality. No association was observed among those with no chronic conditions (Table 5).”

**Reviewer #2: The authors were generally responsive to reviewer comments. I have a few additional questions and comments for their consideration.**

A: Once again, thank you for your useful feedback. Below you can find a point-to-point answer to your questions and comments.

**1. I disagree with the authors that the HBS varies "slightly" from HBI as there are a handful of differences that could each be considered substantial. I think the methods could simply cite the HBS applied by Hu et al and the HBI could be left to the discussion.**

A: Thank you for your comment. We have considered your suggestions as follows:

In the Methods section, we have only cited the HBS used by Hu et. al. as follows:

“A Healthy Beverage Score (HBS) was previously described by Hu et. al. [12]. Based on the HBS, we built a 7-item HBS modifying its cut-off points to fit the beverage consumption of a representative sample of the Spanish adult population.”.

As suggested, the HBI was only cited in the Discussion section:

“In a previous study, a 10-item Healthy Beverage Index was constructed using an a priori approach based on the US recommendations for beverage consumption [11]. Based on this index, Hu et al. described the HBS as more suitable for use in large epidemiological studies [12]. The HBS excluded water consumption as well as two items on total energy from beverages and calculations of daily fluid intake. In our study, the same scoring weights (from 1 to 4) were maintained for all items in the HBS. However, in contrast to Hu et al, we considered both no alcohol consumption and moderate alcohol consumption as healthy. Additionally, we also modified the HBS cut-off points of the items to fit with the beverage consumption of the Spanish adult population. As a result, the HBS used in this study retained the same items as originally described by Hu et al, as well as the relative weight of the items.”

**2. The authors' response to my question about the scoring rationale suggests that had there been a wider distribution of intake for either fruit juice or artificially sweetened beverages then they would have been scored similarly to the other items. It should be noted in the methods that lack of consumption of these beverages is the reason for the difference in scoring.**

A: Thank you. The remark of the reviewer is explained in the new methods section as you can see below:

“The scoring of fruit juices and artificially sweetened beverages was decided on the basis of the lack of a wide range of consumption, and to maintain the relative weight of these items in the score, as previously described by Hu et al [15].”

**3. No where in the methods or tables do the authors define alcohol as grams of ethanol (rather than grams of alcoholic beverages). Given that all other beverages are defined based on the total beverage, and not just a component, I would expect the authors to add this detail.**

A: We have added the following in the Methods section:

“Regarding beverages, a total of 14 digitized photos and 23 household measurements were used to later estimate beverage consumption in milliliters per day. In addition, for alcoholic beverages, the consumption of ethanol in grams per day was calculated using Spanish food composition tables [14].”

[14] Ferrán A, Zamora R, Cervera P, Centre d’Ensenyament Superior de Nitrició i Dietètica (CESNID). (2004) Tablas de composición de alimentos del CESNID. Barcelona: Edicions Universitat de Barcelona.

Also, we have specified that alcohol consumption was measured in grams of ethanol per day in Table 3 footnote.

**4. The authors now use age the underlying time metric but include age as a confounder in the model. Though it wouldn't impact HRs much (if at all), I am unsure if this is a typo in the tables or in fact how the model was constructed. Also, it should be added to the footnotes that age is the underlying time metric as model 1 does account for age if this is the case.**

A: Thank you for this remark. Age was considered both, as an underlying time metric and the analyses were additionally adjusted for age as a confounder. To clarify this point, we have added that age was the underlying time metric in footnotes for tables and figures.

**5. The authors observe an inverse association among those with a chronic disease but not among those without. In line with this finding, there is an inverse association in older but not younger individuals. Given, the wide age range in this study, did the authors look at how the leading causes of deaths differed in these subgroups? Could it be that the HBS is important for chronic disease-specific death only? This is alluded to in the discussion as the potential mechanism discussed are related to chronic disease. The subgroup analyses are also somewhat at odds with the interpretation of HBS being inversely associated with "premature" mortality or mortality in the "general population".**

A: Thank you for your interest in the in-depth interpretation of the results and for your contributions. We do not have enough evidence to state that there is an inverse association in older but not younger individuals. We agree with the idea that the interaction observed according to the presence of chronic conditions deserves further research as expressed in the new version of the manuscript. Unfortunately, the number of events we had did not allow us to study different causes of death that could be an interesting topic in the future.

**6. The abstract and discussion state "a one-quarter reduction in total mortality" this is misleading as results in this analysis are relative and not absolute risk reductions.**

A: Thank you. We have toned down the reporting of results and conclusions to take into account absolute versus the relative risk. We think we have temper the whole manuscript.
